# Sex differences in cardiovascular epigenetics—a systematic review

**DOI:** 10.1186/s13293-018-0180-z

**Published:** 2018-05-23

**Authors:** Robin J. G. Hartman, Sarah E. Huisman, Hester M. den Ruijter

**Affiliations:** Laboratory of Experimental Cardiology, UMC Utrecht, Utrecht University, Utrecht, The Netherlands

**Keywords:** Cardiovascular, Epigenetic, Sex, Gender, Stratification, DNA methylation, Systematic review

## Abstract

**Background:**

Differences in cardiovascular diseases are evident in men and women throughout life and are mainly attributed to the presence of sex hormones and chromosomes. Epigenetic mechanisms drive the regulation of the biological processes that may lead to CVD and are possibly influenced by sex. In order to gain an overview of the status quo on sex differences in cardiovascular epigenetics, we performed a systematic review.

**Materials and methods:**

A systematic search was performed on PubMed and Embase for studies mentioning cardiovascular disease, epigenetics, and anything related to sex differences. The search returned 3071 publications to be screened. Primary included publications focused on cardiovascular and epigenetics research. Subsequently, papers were assessed for including both sexes in their studies and checked for appropriate sex stratification of results.

**Results:**

Two independent screeners identified 75 papers in the proper domains that had included both sexes. Only 17% (13 papers out of 75) of these publications stratified some of their data according to sex. All remaining papers focused on DNA methylation solely as an epigenetic mechanism. Of the excluded papers that included only one sex, 86% (24 out 28) studied males, while 14% (4 out of 28) studied females.

**Conclusion:**

Our overview indicates that the majority of studies into cardiovascular epigenetics do not show their data stratified by sex, despite the well-known sex differences in CVD. All included and sex-stratified papers focus on DNA methylation, indicating that a lot of ground is still to gain regarding other epigenetic mechanisms, like chromatin architecture, and histone modifications. More attention to sex in epigenetic studies is warranted as such integration will advance our understanding of cardiovascular disease mechanisms in men and women.

**Electronic supplementary material:**

The online version of this article (10.1186/s13293-018-0180-z) contains supplementary material, which is available to authorized users.

## Background

Cardiovascular disease (CVD) is an annual leading cause of mortality across the world. The World Health Organization reported about 17.7 million deaths resulting from CVD in 2015, amounting to a staggering 31% of all the deaths that year [1]. CVD differs in men and women. Macroscopically, men have larger hearts and blood vessels as compared to women [2–4]. Clinically, men tend to develop CVD at a younger age, develop more severe coronary artery disease, and present with heart failure with reduced ejection fraction. Women present with CVD later in life when more comorbidities such as diabetes are present and more often develop non-obstructive coronary artery disease, and they preserve their ejection fraction when heart failure is diagnosed.

The field of epigenetics is rapidly growing, with increasingly more focus and highlight on the link between our epigenetic makeup and CVD etiology and predisposition. As the definition of epigenetics is rather vague and under continuing debate [5], we define epigenetic here as mitotically stable, non-sequence-dependent mechanisms such as DNA methylation and histone modifications that make up the epigenetic landscape. These mechanisms may subsequently influence gene expression, independently of the genetic code. While the amount of research linking epigenetics and CVD is climbing, it is of critical importance that these studies should be stratified according to sex. First, epigenetic mechanisms ensure the inactivation of the second X-chromosome in women, securing dosage compensation of the X-chromosome between men and women [6]. In addition, epigenetic mechanisms control sex-specific gene expression during development in tissue [7]. Furthermore, epigenetic mechanisms set the sex-specific stage for diseases later in life [7]. On top of that, the sex chromosomes contain multiple epigenetic modifiers which are differentially expressed between the sexes, which might influence the autosome in a sex-specific manner [8]. Moreover, steroid sex hormones such as estrogen and testosterone have been shown to affect epigenetic modifications [9, 10]. Another often overlooked reason is the sex-specific impact of the environment on gene expression regulation [7]. To summarize the current knowledge on sex differences in cardiovascular epigenetics, we performed a systematic review of available literature.

## Methods

### Search criteria and screening process

**Fig. 1:**
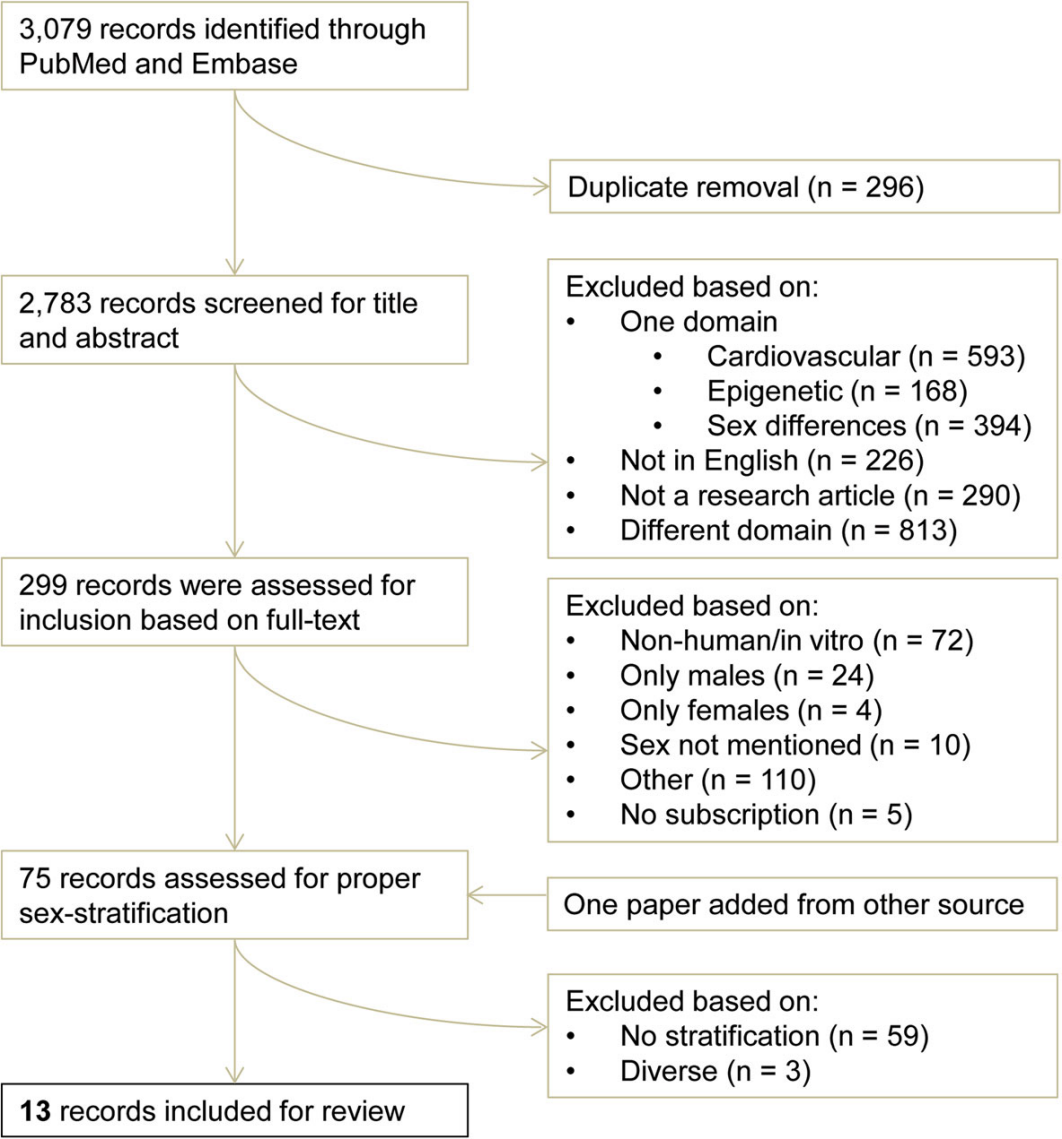
Workflow of the systematic search and review. A flow-chart regarding the systematic search and review process is shown, starting with the number of papers found in the top, leading to the number of papers included in the bottom

A systematic search was performed on 14 May, 2017, on PubMed (https://www.ncbi.nlm.nih.gov/pubmed) and Embase (https://embase.com/#search). The exact search strings used can be found in Additional file 1: Table S1. The screening process is depicted in Fig. 1. Two independent researchers (RH and SH) screened all papers found. Whenever there was disagreement about inclusion of papers, a third individual was involved, and the paper was discussed until consensus was reached. Duplicates were removed, and a title/abstract screen was performed to assess primary inclusion based on CVD and epigenetics. Subsequently, a full-text screen was performed to assess whether studies included both men and women and if interaction for sex was tested and whether or not sex-stratified results were presented. Full-texts were also double checked for the proper cardiovascular domain, including papers reporting on CVD (such as heart failure and atherosclerosis), but excluding papers reporting on diseases such as kidney disease. We included papers reporting on cardiovascular function, as well as papers reporting on CVD risk factors that belong to the Framingham Risk Score: age, gender, lipid levels, smoking, blood pressure, and adding BMI, metabolic syndrome, and diabetes. Papers focusing on non-modifiable risk factors such as ethnicity and family history were excluded. We excluded papers reporting on pregnancy-related epigenetics. Telomere length was not considered an epigenetic mechanism in this review. Data extracted for Table 1 included the CVD (risk factor) studied, the epigenetic mechanism, technique for measuring the epigenetic state, size study population and sex, tissue, the gene studied, and the association.

**Table 1:** Summary of included papers performing proper sex stratification in the same order as mentioned in the main text

Year	First author	CVD/risk factor	Epigenetics	Sample size (% male)	Technique	Tissue	Gene (if applicable)	Association	Ref.
2017	Mendelson	BMI	DNA methylation	3743 (48%)	Illumina Infinium 450K	Whole blood	*LGALS3BP* (unannotated CpG)	Stronger for ♂	[12]
2011	Cash	Factors associated with obesity and CVD	DNA methylation	355 (25%)	Bisulphite into pyrosequencing	Lymphocytes	Global	More methylation in ♂	[13]
2013	Guay	Blood lipid levels	DNA methylation	98 (62%)	Bisulphite into pyrosequencing	Leukocytes	*CETP*	Stronger for ♂	[14]
2014	Guay	Plasma lipid levels	DNA methylation	98 (62%)	Bisulphite into pyrosequencing	Leukocytes	Multiple		[15]
2013	Zhang	Metabolic syndrome	DNA methylation	517 (41%)	EpiTYPER	Leukocytes	*FABP3*		[16]
2013	Johansson	Aging	DNA methylation	421 (not specified)	Illumina Infinium 450K	Leukocytes	Epigenome-wide		[17]
2016	Horvath	Aging	DNA methylation	4535 (35%)	Illumina Infinium 450K	Blood/saliva/brain	Epigenome-wide		[18]
2014	Soriano-Tárrago	Ischemic stroke	DNA methylation	485 (62%)	LUMA	Whole blood	Global	Hypomethylation for ♂	[19]
2017	Lin	Ischemic stroke	DNA methylation	556 (48%)	Bisulphite into pyrosequencing	Whole blood	*MMP2*	Only for ♂	[20]
2012	Talens	Myocardial infarction	DNA methylation	248 (52%)	Mass spectrometry	Leukocytes	*INS*, *GNASAS*	Only in MI samples of ♀	[21]
2013	Jiang	Coronary heart disease	DNA methylation	72 (50%)	Bisulphite into pyrosequencing	Whole blood	*PLA2G7*	Only for ♀	[22]
2016	Guo	Coronary artery disease	DNA methylation	64 (56%)	Methylation-specific PCR	Whole blood	*PTX3*	Only for ♂	[23]
2014	Zhang	CVD mortality	DNA methylation	3588 (44%)	MALDI-TOF	Whole blood	*F2RL3*	Stronger for ♂	[24]

*BMI* body mass index, *Ref* reference

## Results

### Systematic search

The combined search returned 3071 publications (Fig. 1). After duplicate removal, 2783 publications were left, which were then screened for their title and abstract, yielding 299 papers for the full-text screening. The 2484 excluded papers contain only one domain (cardiovascular, epigenetics, or sex differences) or no domain at all, are reviews/commentaries/book chapters, or are not published in English. Of the 299 primary included papers in cardiovascular epigenetics, 74 publications had included both men and women (Fig. 1). Of the 225 secondary excluded papers, 28 included only one sex and 10 papers did not mention the sex. Of the papers including one sex, 86% (24 out of 28) included only males and 14% (4 out of 28) included only females. We added one extra publication found via other sources to these 74 papers, leading to 75 papers. Startlingly, only 13 papers stratified their data for sex of the 75 publications that included males and females. In the end, these 13 papers were included for the review, all summarized in Table 1. We also excluded three papers from the 75 publications based on diverse criteria for not properly stratifying, although still showing male and female data (Fig. 1).

### Review of included papers

All of the included papers look at DNA methylation as an epigenetic mechanism in either blood or leukocytes, with a variety of techniques, such as arrays or bisulphite treatment followed by pyrosequencing (Table 1). Therefore, a small intermezzo about DNA methylation is in place. In mammals, DNA methylation almost exclusively indicates DNA cytosine methylation. Cytosines targetable for methylation are practically always followed by a guanine, giving rise to the CpG dinucleotide [11]. Promoter areas of genes are enriched for CpG dinucleotides, and methylation of promoter regions enriched for CpGs is associated with repression of transcription [11]. DNA methylation plays a key role in maintenance of cell identity as well as in differentiation of cells by repressing transcription of genes which are obsolete in specific lineages [7].

The following section will review the included papers.

### Sex differences in the epigenetics of cardiac risk factors

Seven papers looked at one or more risk factors for CVD. All of the seven papers report at least one difference found in epigenetic markers and/or their associations between men and women. Mendelson et al. looked at whole-blood DNA methylation and its relation to BMI [12]. Among 135 discovered CpGs, they report a significant sex interaction for an unannotated CpG positioned closest to the *LGALS3BP* gene. This CpG was also found in replication cohorts, with larger regression coefficients and smaller *p* values reported in men as compared to women.

Cash et al. show that LINE-1 methylation, used as a proxy for global DNA methylation, is higher in men as compared to women [13]. Furthermore, stratified analyses show LDL and HDL relationships with LINE-1 methylation only in men and a relationship between BMI and LINE-1 methylation only in women.

Guay et al. studied *CETP* and *LPL* promoter methylation and its relationship to blood lipid levels [14]. They report for both sexes negative association between *CETP* promoter methylation and LDL cholesterol levels (*r* < − 0.32; *p* < 0.05) and for men as well associations between *CETP* promoter methylation and HDL-C (*r* = − 0.36; *p* = 0.006), HDL-triglyceride levels (*r* = 0.59; *p* < 0.001), and HDL particle size (*r* = − 0.44, *p* = 0.019). Methylation of *CETP* and *LPL* promoters tend to be higher in women as compared to men.

Another paper by Guay et al. investigated epipolymorphisms in lipoprotein genes and their relationship to plasma lipid levels [15]. *ABCG1* showed higher methylation in men as compared to women (*p* = 0.007), while a CpG in *PLTP* showed higher methylation in women as compared to that in men (*p* = 0.025). Higher methylation of CpGC3 in *ABCG1* in women was associated with lower TG levels, while in men this methylation was associated with age, a larger waist circumference, and lower total cholesterol levels. Higher methylation of *LIPC*-CpGA2 in men was associated with lower HDL-C and TG levels, while a positive trend was noticeable for TG levels in women. They also show that epipolymorphisms in *ABCG1*, *LIPC*, *PLTP*, and *CETP* contributed to variations found in plasma lipid levels, with stronger associations in men, independently of traditional predictors.

Zhang et al. examined DNA methylation in different CpGs of *FABP3* and its relationship to insulin, lipids, and cardiovascular phenotypes [16]. They found that multiple different CpGs within *FABP3* were influenced by sex, indicating that specific sites within genes are prone to sex differences, which might relate differently to disease outcome.

Johansson et al. looked into the relationship of aging and DNA methylation in white blood cells [17]. They report significant interactions between aging and sex for 163 CpGs, of which 152 CpGs are located on the X-chromosome. It is not reported which CpG shows a significant sex interaction.

Horvath et al. looked at epigenetic aging rates in different tissues by using DNA methylation [18]. They show that men have higher epigenetic aging rates than women in blood, saliva, and brain tissue. Furthermore, epigenetic aging rates were also associated with CVD risk factors, but not with CVD outcomes.

### Sex differences in the epigenetics of cardiovascular disease and mortality

Five papers looked directly at CVD outcomes, of which ischemic stroke, myocardial infarction, coronary heart disease and coronary artery disease, and one included paper investigated cardiovascular mortality.

Soriano-Tárrago et al. studied global DNA methylation of ischemic strokes subtypes [19]. No distinct differences were found between the different ischemic stroke subtypes regarding DNA methylation, but global DNA hypomethylation was described in men as compared to women over all samples.

Lin et al. looked at methylation of *MMP2* in ischemic stroke [20]. They show that patients suffering from ischemic stroke have lower levels of *MMP2* methylation at multiple CpGs as compared to healthy controls. This association was stronger in men (*p* values ranging from 0.001–0.056) as compared to women (*p* values ranging from 0.051–0.354).

Talens et al. looked into DNA methylation of loci sensitive to prenatal environment and its relationship to myocardial infarction [21]. DNA methylation of *INS* and *GNASAS* in leukocytes were higher in myocardial infarction in females (*INS*: *p* = 0.002; *GNASAS*: *p* = 0.001), with no associations found in men. This indicates that the sex-specific stage for disease predisposition might be set very early in life.

Jiang et al. focused on *PLA2G7* promoter methylation and its relation to coronary heart disease [22]. They describe a signification association between *PLA2G7* promoter methylation and coronary heart disease in women (*p* = 0.003), which was not found in men (*p* = 0.096). Moreover, this association was independent of age, smoking, hypertension, and diabetes. The *PLA2G7* promoter methylation was also associated with total cholesterol levels, triglyceride levels, and apolipoprotein B in women, but not in men.

Guo et al. investigated promoter DNA methylation of *PTX3* in coronary artery disease [23]. They show that lower methylation of *PTX3* is associated with higher PTX3 plasma levels. In men only, lower *PTX3* methylation was associated with a higher neutrophil to lymphocyte ratio (*r* = − 0.58, *p* = 0.002).

Zhang et al. studied methylation of *F2RL3*, an epigenetic biomarker of smoking exposure, and its relationship to mortality outcomes [24]. They found that lower methylation intensity of *F2RL3* correlated strongly with mortality outcomes, as well as cardiovascular mortality. The associations found were all much stronger in men as compared to women.

## Discussion

In this systematic review of over 3000 publications found by the search, we highlight the insufficient amount of sex stratification performed in cardiovascular epigenetic research and reviewed the available stratified data according to CVD risk factors and their etiology.

### Sex stratification in cardiovascular epigenetics

Our overview indicates that the majority of studies into cardiovascular epigenetics do not show their data stratified by sex, despite the well-known sex differences in CVD. Moreover, from the papers describing only one sex, 86% studied men and 14% studied women suggesting that the underrepresentation of women is an ongoing phenomenon, while the knowledge on the female pathology of CVD is limited. Women have not been equally represented in clinical trials for CVD as compared to men [25], as women have been excluded to protect them from any adverse drug reactions after the thalidomide tragedy in the 1950s. On top of that, women are often excluded because of fluctuating hormone levels starting from puberty, which complicate the implementation of acquired data. Another reason why women have been underrepresented is that women develop CVD later in life, while suffering from multiple comorbidities, leading to exclusion of studies. In larger genome-wide association studies, sex chromosomes are often dismissed, as analysis of the sex chromosomes is laborious and requires different algorithms as compared to autosomal chromosomes [26]. Furthermore, most animal studies do not have an equal representation of the sexes [27], while it has been demonstrated that a large proportion of mammalian traits are influenced by sex in wild-type and mutant animals [28].

Evidently, our results (Fig. 1) point to the scarcity of sex stratification in cardiovascular epigenetics. Of the 75 studies that described both sexes to be present in their data, only 13 publications had performed sex stratification in their studies. Often, associations between specific epigenetic markers and cardiovascular phenotypes were found, while only holding true in one of the sexes. Papers that were included primarily, but excluded during the second round, often immediately adjust for the effect of sex, as sex is known to have a major impact on epigenetics. Consequently, the lack of proper stratification can lead to the overlooking of sex-specific epigenetic effects.

### Global DNA methylation

Differences in global DNA methylation profiles between the sexes are an area of open debate. Some of the included studies had conflicting conclusions about DNA methylation. For example, Cash et al. demonstrated that there is a higher global DNA methylation in males [13], while Soriano-Tárraga et al. showed that hypomethylation is associated with the male sex [19]. The latter paper looked at methylation in whole blood, while the first studied DNA methylation in lymphocytes, which might potentially explain some of the differences seen here, as the epigenetic makeup is tissue-dependent. Johansson et al. also pointed out a lower median autosomal DNA methylation in men as compared to women, while mean levels of DNA methylation did not differ [17]. This indicates that the distribution of methylation on the autosome might be subject to sex differences, as seen for the sex chromosomes. Interestingly, men showed a higher degree of epigenetic aging based on DNA methylation in three out of the three tissues as compared to women [18]. This points towards a general epigenetic mechanism in different tissues that is influenced by sex. However, zooming in on specific genes affected by the differential methylation might provide more information on epigenetic mechanisms that differ between men and women. The included papers point towards general genome-wide and specific regulatory mechanisms influenced by sex.

### Epigenetic mechanisms

**Fig. 2:**
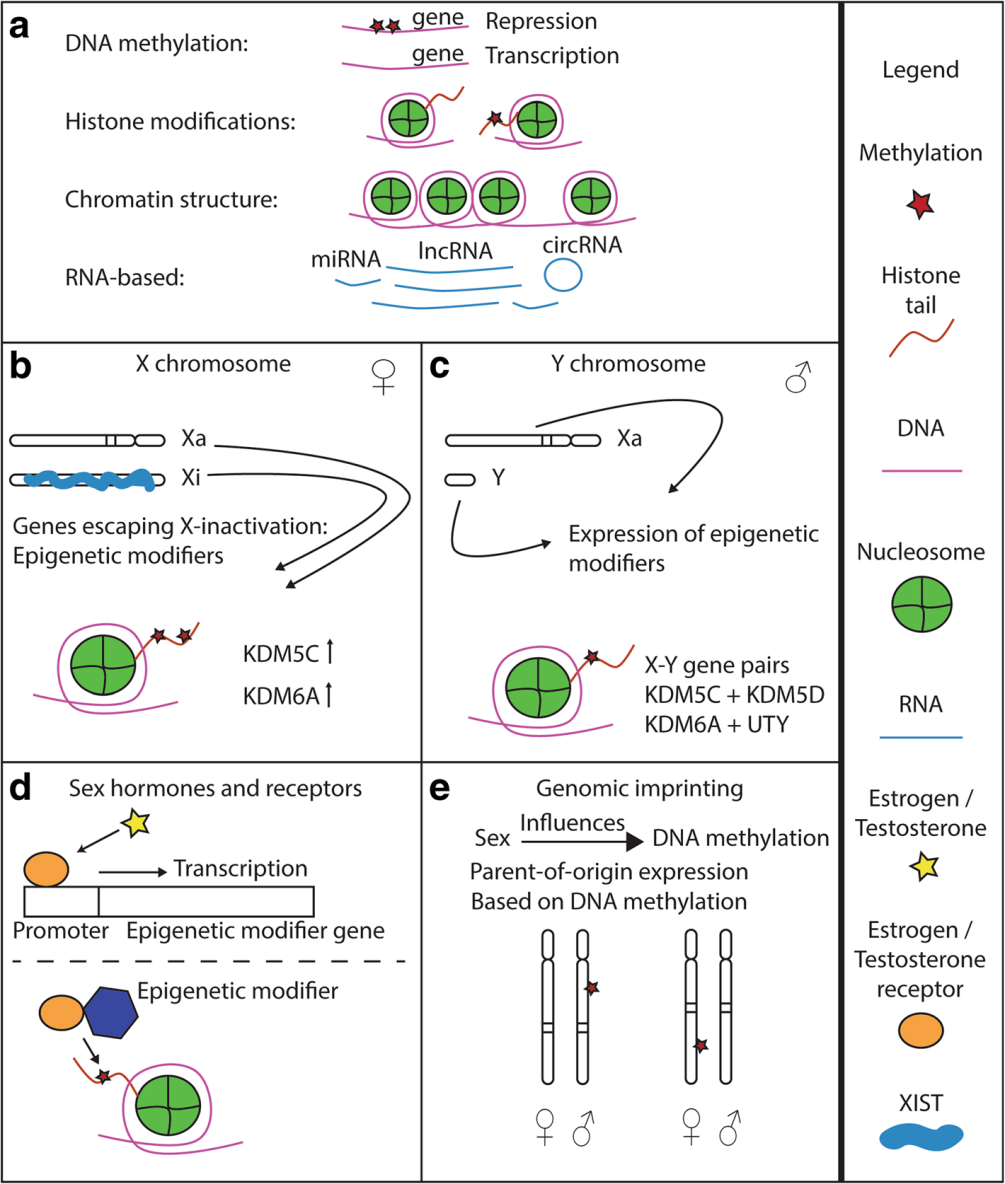
Epigenetic mechanisms and sex. **a** The epigenetic mechanisms as set forth in the main text are schematically depicted here, with a legend on the right. **b** Possible ways in which sex influences epigenetic mechanisms. Higher expression of X-chromosomal genes that escape inactivation in women might influence epigenetic markers differently as compared to men. **c** The Y chromosome contains different epigenetic modifiers which might influence the autosome in men, but not in women. **d** Sex hormones and their receptors can influence the gene expression of epigenetic modifiers, as well as interact with epigenetic modifiers. **e** Genomic imprinting is an example in which sex influences DNA methylation, as particular maternal or paternal alleles are differentially methylated

We observed that DNA methylation was the only highlighted epigenetic mechanism in association with cardiovascular phenotypes (see Fig. 2a for a schematic on common epigenetic mechanisms). One reason could be that it is less costly and labor-intensive to investigate genome-wide DNA methylation on widely available arrays, as compared to investigating other epigenetic mechanisms that are not yet available on standard platforms, such as chromatin confirmation capture (3C, 4C, Hi-C) sequencing, assay for transposase accessible (ATAC) sequencing, and chromatin immunoprecipitation (ChIP) sequencing. Nevertheless, the sample size of the included studies ranges from 64 to well over 3000, although the larger epigenome-wide association studies are scarce. Future advances in ChIP sequencing should allow for larger cohort histone modification studies as well. Another epigenetic feature that may be studied on a broader scale in the near future is chromatin organization, with assays such as Hi-C sequencing or ATAC sequencing.

### Influence of sex on epigenetic mechanisms

There are multiple mechanisms by which sex might directly influence cardiovascular epigenetics (see Fig. 2b-e): (1) The increased expression of X-chromosomal escape genes in women, of which some target epigenetic modifications; (2) the expression of non-pseudo-autosomal Y-chromosomal epigenetic modifiers in men; (3) the (non-)genomic effect of steroid hormones and their receptors on epigenetic regulators, such as DNA methylation enzymes, histone modifiers, and miRNAs; and (4) genomic imprinting, leading for example to DNA methylation of either maternal or paternal alleles. The effect of sex on epigenetic marks might subsequently lead to changes in gene expression, culminating in sex differences in CVD.

### Influence of the non-coding genome

The action of microRNAs is considered to be an epigenetic mechanism. Differential miRNA expression is very likely to exist between the sexes, as the X-chromosome contains about 10% of miRNAs harbored in the human genome. This higher density of miRNAs on the X-chromosome as compared to autosomes is noted in other mammalian species as well [29]. This suggests that the X-chromosome has an indispensable role in miRNA-mediated regulation of gene expression, and it might be interesting to find out if any of these miRNAs associate with CVD. A recent review has focused on the potential role of sex-biased miRNAs in the etiology of heart failure with preserved ejection fraction, explaining how sex also affects this epigenetic feature in a syndrome presenting with differential prevalence between the sexes [30]. In addition to miRNAs, the regulatory role of long non-coding RNAs (lncRNA) and circular RNAs should be looked at in a sex-specific way. One of the most studied lncRNAs is XIST, which is crucial for establishing X-chromosomal gene expression dosage compensation in women [6].

### Sex stratification and statistical power

Our systematic search revealed that sex stratification is rare in cardiovascular epigenetics, even when sex terms were incorporated in the search string to look for sex-specific epigenetic mechanisms. When sex terms were excluded from the search string, we were returned with twice the number of papers, indicating that the actual definite amount of sex stratification done in cardiovascular epigenetic studies could be even lower. Studies with small sample sizes are limited by their statistical power to detect sex differences, resulting in possible exclusion of sex stratification during their experimental design. CVD with a low prevalence, such as specific gene mutation cardiomyopathies, might be difficult to study in a sex-specific manner, as statistical power will be problematic to acquire. An increase in sample size is not always easy to obtain for power, which warrants different teams to collaborate. Large data sets that permit answers to multiple questions, which can be built as a joint force, will grant more power for testing interactions. Meta-analyses of already existing datasets might give insights to the power issue as well. For future studies on CVD, uniformity in arrays and techniques will aid detection of sex interaction in epigenome-wide designs. Another option might be to let journals require authors to present sex-stratified data in the supplementary material (regardless of significant interactions). Some studies might have investigated the interaction of sex and epigenetic processes, but did not report them in their results. It is valuable to mention a tested but not deviating sex interaction as it points towards similar biology between men and women. A number of excluded studies which did not stratify accordingly for sex mentioned the importance of sex as a variable in epigenetic research and subsequently adjusted for the effect of sex in their model, potentially missing out on mechanisms and drug targets that are sex-specific. Although the knowledge on the importance of sex in biomedical sciences is emerging, a declaration of sex-interaction testing in journals would be incredibly helpful for a wide-scale and fair investigation into the bona fide differences between epigenetic mechanisms in the sexes. The National Institutes of Health in the USA already requires investigators to account for sex and gender, and more measures are being taken to hold scientists more accountable for implementing sex as a biological variable [31].

### Limitations

A broad overview of microRNA differences between the sexes was not performed, as the search string limited the amount of papers found on microRNAs, e.g., RNA interference is a subheading under the MeSH term “epigenesis,” but not every paper on microRNAs might be indexed in this fashion. A meta-analysis on transcriptomic differences and thus as well expression of microRNAs between the sexes in different types of cardiovascular tissues would be of value to address this. Although we did not include a publication date criteria, all of the included papers had been published in the last decade, demonstrating that more attention has been given to proper sex stratification recently. However, the *status quo* is still far from ideal. A limitation of our study is that we limited our search to two databases and did not include pre-print archives or other databases. Therefore, we cannot make statements on the total body of literature available in all systems.

## Conclusion

Our systematic review highlighted the lack of stratification in cardiovascular epigenetics, with only 13 out of 75 included publications stratified according to sex. Most of the papers that were excluded regressed out the effect(s) of sex in their studies. Together, our review underscores the acute need to investigate the effects of sex on epigenetic mechanisms as current research reflects the strong influence of sex on epigenetic regulatory processes.

## Additional file

Additional file 1: **Table S1.** Search strings used for PubMed and Embase. (DOCX 14 kb)

## Abbreviations

CpG: Cytosine guanine dinucleotide
CVD: Cardiovascular disease
