# Supplementary material for: Sex differences in cardiovascular epigenetics—a systematic review
Source: Biol Sex Differ. 2018 May 23;9:19. doi: 10.1186/s13293-018-0180-z (PMC5966883; doi:10.1186/s13293-018-0180-z)
Supplement: Supplementary file 1 — Table S1. Search strings used for PubMed and Embase. (DOCX 14 kb) [file 13293_2018_180_MOESM1_ESM.docx]

Additional file 1: Table S1 Search strings used for PubMed and Embase.

| Database | PubMed | Embase |
| --- | --- | --- |
| Date search | 14-05-2017 | 14-05-2017 |
| General | Only English language | Only English language |
| Filters / limitations | Filters: Classical Article, Clinical Study, Clinical Trial, Clinical Trial, Phase I, Clinical Trial, Phase II, Clinical Trial, Phase III, Clinical Trial, Phase IV, Comparative Study, Journal Article, Randomized Controlled Trial, Dataset, Controlled Clinical Trial, Corrected and Republished Article, Meta-Analysis, Introductory Journal Article, Humans. | Limitations: ([article]/lim AND [humans]/lim) |
| Cardiovascular | "Cardiovascular Diseases"[Mesh] OR cardiovascular diseases[tiab] OR cardiovascular disease[tiab] OR "cardiology"[MeSH Terms] OR cardiology[tiab] OR heart failure[tiab] OR myocardial infarction[tiab]) | ('cardiovascular disease'/exp OR (cardiovascular AND disease:ti,ab) OR (cardiovascular AND diseases:ti,ab) OR 'cardiology'/exp OR cardiology:ti,ab OR 'heart failure'/exp OR (heart AND failure:ti,ab) OR 'heart infarction'/exp (heart AND infarction:ti,ab)) |
| Sex/gender | ("sex"[MeSH Terms] OR "gender identity"[MeSH Terms] OR "sex characteristics"[MeSH Terms] OR "female"[MeSH Terms] OR "male"[MeSH Terms] OR gender[tiab] OR sex[tiab] OR male[tiab] OR female[tiab] OR males[tiab] OR females[tiab] OR "menopause"[MeSH Terms] OR menopause[tiab] OR "postmenopause"[MeSH Terms] OR postmenopause[tiab] OR "premenopause"[MeSH Terms] OR premenopause[tiab]) | ('gender and sex'/exp OR gender:ti,ab OR sex:ti,ab OR 'gender identity'/exp OR (gender AND identity:ti,ab) OR 'sexual development'/exp OR (sexual AND development:ti,ab) OR 'female'/exp OR females:ti,ab OR female:ti,ab OR 'male'/exp OR male:ti,ab OR males:ti,ab OR 'menopause'/exp OR menopause:ti,ab OR 'postmenopause'/exp OR postmenopause:ti,ab OR 'premenopause'/exp OR premenopause:ti,ab) |
| Outcome | ("epigenesis, genetic"[MeSH Terms] OR "epigenomics"[MeSH Terms] OR epigenetics[tiab] OR epigenetic[tiab] OR epigenesis[tiab] OR epigenomics[tiab] OR epigenomic[tiab] OR "histones"[MeSH Terms] OR histones[tiab] OR "chromatin"[MeSH Terms] OR "sex chromatin"[MeSH Terms] OR chromatin[tiab] OR sex chromatin[tiab] OR "heterochromatin"[MeSH Terms] OR heterochromatin[tiab] OR "euchromatin"[MeSH Terms] OR euchromatin[tiab] OR euchromatins[tiab] OR heterochromatins[tiab] OR chromatins[tiab] OR sex chromatins[tiab]) | 'genetic epigenesis'/exp OR (genetic AND epigenesis:ti,ab) OR 'epigenetics'/exp OR epigenetics:ti,ab OR 'histone'/exp OR histone:ti,ab OR 'chromatin'/exp OR chromatin:ti,ab OR 'x chromatin'/exp OR 'y chromatin'/exp OR histones:ti,ab OR chromatins:ti,ab OR (x AND chromatins:ti,ab) OR (y AND chromatins:ti,ab) OR (y AND chromatin:ti,ab) OR (x AND chromatin:ti,ab) OR 'heterochromatin'/exp OR heterochromatins:ti,ab OR heterochromatin:ti,ab OR 'euchromatin'/exp OR euchromatins:ti,ab OR euchromatin:ti,ab |
| Search results | 1949 items | 1130 items |
